# Identification of stage‐specific blood biomarkers for early diagnosis of Alzheimer’s disease through RNA sequencing analysis

**DOI:** 10.1002/alz.085323

**Published:** 2025-01-09

**Authors:** Akiko Yamakawa, Mutsumi Suganuma, Risa Mitsumori, Shumpei Niida, Kouichi Ozaki, Daichi Shigemizu

**Affiliations:** ^1^ National Center of Geriatrics and Gerontology, Obu, Aichi Japan; ^2^ National Center for Geriatrics and Gerontology, Obu, Aichi Japan; ^3^ Hiroshima University Graduate School of Biomedical and Health Sciences, Hiroshima, Hiroshima Japan; ^4^ RIKEN Center for Integrative Medical Sciences, Yokohama, Kanagawa Japan

## Abstract

**Background:**

The detailed mechanism of Alzheimer’s disease (AD) has not been fully elucidated, and a comprehensive gene expression analysis of the entire process leading up to the onset of AD has never been conducted on a large scale.

**Method:**

We performed RNA‐seq analysis of 1227 blood samples consisting of 424 AD, 543 mild cognitive impairment (MCI), and 260 cognitive normal (CN) subjects, and examined differentially expressed genes (DEGs) between CN and MCI (CN‐MCI), and between MCI and AD (MCI‐AD). Pathway analysis of DEGs were performed and subsequently retrospective prediction models were constructed using the enriched genes within these pathways.

**Result:**

A total of 883 and 1,169 statistically significant DEGs were identified in CN‐MCI and MCI‐AD, respectively. Pathway analyses using these DEGs and subsequent construction of risk prediction models led to the identification of 6 and 29 representative genes, achieving area under the curves of 0.792 (95% confidence interval: 0.768‐0.853) for CN‐MCI and 0.781 (95% confidence interval: 0.712‐0.799) for MCI‐AD. Furthermore, protein–protein interaction network analysis using the representative genes revealed key roles of ribosomal genes and phagosomes in the progress of MCI, while immune‐related genes were involved in the progression of AD.

**Conclusion:**

Our findings suggest that the onset of AD may be associated with gene expression changes in the immune system, protein processing and cell cycle, following observed alterations in gene expression within ribosomes and phagosomes during the MCI stage. Given the effectiveness of delaying MCI progression in preventing AD, the regulation of ribosomal expression and phagocytosis emerges as potential biomarkers.

